# Isotype Specific Assembly of B Cell Antigen Receptors and Synergism With Chemokine Receptor CXCR4

**DOI:** 10.3389/fimmu.2018.02988

**Published:** 2018-12-18

**Authors:** Palash C. Maity, Moumita Datta, Antonella Nicolò, Hassan Jumaa

**Affiliations:** Institute of Immunology, Ulm University, Ulm, Germany

**Keywords:** B cell antigen receptor (BCR), Chemokine receptor 4 (CXCR4), Nanoclusters, Cytoskeleton, B cell malignancies

## Abstract

Expression of the membrane-bound form of the immunoglobulin (Ig) as part of the antigen receptor is indispensable for both the development and the effector function of B cells. Among five known isotypes, IgM and IgD are the common B cell antigen receptors (BCRs) that are co-expressed in naïve B cells. Despite having identical antigen specificity and being associated with the same signaling heterodimer Igα/Igβ (CD79a/CD79b), IgM and IgD-BCR isotypes functionally differ from each other in the manner of antigen binding, the formation of isolated nanoclusters and in their interaction with co-receptors such as CD19 and CXCR4 on the plasma membrane. With recent developments in experimental techniques, it is now possible to investigate the nanoscale organization of the BCR and better understand early events of BCR engagement. Interestingly, the cytoskeleton network beneath the membrane controls the BCR isotype-specific organization and its interaction with co-receptors. BCR triggering results in reorganization of the cytoskeleton network, which is further modulated by isotype-specific signals from co-receptors. For instance, IgD-BCR is closely associated with CXCR4 on mature B cells and this close proximity allows CXCR4 to employ the BCR machinery as signaling hub. In this review, we discuss the functional specificity and nanocluster assembly of BCR isotypes and the consequences of cross-talk between CXCR4 and IgD-BCR. Furthermore, given the role of BCR and CXCR4 signaling in the development and survival of leukemic B cells, we discuss the consequences of the cross-talk between CXCR4 and the BCR for controlling the growth of transformed B cells.

## Introduction

B-lymphocytes (B cells) are central to the mammalian humoral immune response, as they produce and secrete immunoglobulins (Igs), also known as antibodies that contribute to neutralization, fixation, and clearance of pathogens. Besides the secreted form of Igs, B cells also express a membrane-bound form of Ig (mIg) as part of the B cell antigen receptor (BCR), which is indispensable for B cell differentiation, survival, and activation (1–3). While it is unclear how in the absence of foreign antigens BCR-derived signals regulate selection and survival of B cells throughout development, it is evident that binding of foreign antigen to mature B cells triggers BCR-dependent proliferation and differentiation of the mature B cells into antibody secreting plasma cells or memory B cells (1, 3–5). Each B cell expresses a unique BCR specificity as a result of the random rearrangement of the *IG*-gene segments in the course of early developmental stages (6–8). This process generates a highly diverse pool of naïve B cells carrying arrays of specificities, which could theoretically distinguish >10^14^ different non-self molecular monograms or antigens (9, 10). Upon antigen encounter, the selected BCR specificities are further modified through the process of somatic hypermutation (SHM) within the germinal center (GC) (11–14), thereby resulting in optimized antibodies against invading pathogens.

The unique antigen binding specificity of an antibody is determined by the combination of its heavy chain (HC) and light chain (LC) variable domains (V_H_ and V_L_, respectively), produced by recombination of the variable (V), diverse (D), and joining (J) segments of the *IG* gene. A pair of recombination activating genes called RAG1 and RAG2 catalyze the V(D)J recombination during the development of B cells (15). Once generated, the recombined and selected V(D)J rearrangements provide unique antigen binding specificity to the respective B cell (16–19). By alternative splicing of pre-mRNA or class-switch recombination (CSR), a recombined VDJ cassette can be expressed as IgM, IgD, IgG, IgA, or IgE isotypes, by using different constant gene segments. Each secretable isotype possesses different neutralization, fixation, and clearance role (20–23). Although the V_H_ and V_L_ regions determine the antigen binding specificity, the constant region of Ig has an important role in fine-tuning the antigen sensing process (20, 22, 23).

In principle, all the five isotypes can be spliced as the membrane-associated mIg form thereby presenting as BCR on the B cell surface (4). During early development, B cells express only IgM-BCR, while IgD is produced later along with IgM by alternative pre-mRNA splicing at mature B cell stages (6, 24, 25). After encountering an antigen, IgM^+^IgD^+^ mature B cells undergo CSR to produce IgG, IgA, or IgE isotypes. Interestingly, B cells do not equally utilize the BCR isotypes. However, the mechanisms regulating this selectivity are not fully understood. For instance, IgA-BCR is relatively common in human but rare in mouse, while IgE-BCR is completely underrepresented in both species (26–28). This might indicate that BCR isotypes possess different affinity for distinct antigens, that they own different signaling capacities or that they are specialized for specific antigen forms (4, 20, 22, 23). In line with these views, the IgG-BCR produces more traction force than IgM-BCR while interacting with membrane-bound antigens, suggesting a specialized role of IgG-BCR to interact with complex or membrane-bound antigens (29, 30). Moreover, the co-existence of IgM and IgD-BCR on naïve recirculating B cells also provokes the hypothesis of a functional difference. However, the specific role of the IgD-BCR remained obscure for a long time. With the advent of cutting edge technology, accumulating evidence points to functional differences between these two BCR isotypes. For instance, it has been found that IgM and IgD-BCRs do differ in antigen sensing, signal commitment, structural flexibility as well as in their nanocluster organization on the plasma membrane (PM) landscape (31–33).

Therefore, it is important to discuss the functional specificities of IgM and IgD-BCRs in light of B cell development (section Altered B cell development), antigen selectivity (section Selective antigen responsiveness), and GC response and affinity maturation (section GC response and affinity maturation). In addition, we explain how nanocluster assembly of different BCR isotypes on mature B cells supports their functional differences (section Characterization of BCR nanoclusters). In light of this isotype-specific segregation, we address the interaction between BCR isotypes and co-receptors as well as the consequences of these processes in B cell activation and B cell-related diseases (section Synchronization effect of chemokine receptor CXCR4).

## Functional Specificity of BCR Isotypes

Since mature naïve B cells express both IgM and IgD-BCR on their surface, it has been proposed that these two BCR isotypes are functionally redundant. Several lines of evidence support this view. First, mIgM and mIgD are generated from alternative splicing of the same pre-mRNA thereby having the same variable (V_H_) region and identical antigen binding specificity. Second, both mIg classes are associated with the Igα/Igß heterodimer (encoded by *CD79A* and *CD79B* genes, respectively), for signal initiation and a plethora of common signaling proteins including BLNK (also known as SLP65) Syk, Lyn, Btk, or PLCγ2 to transmit and integrate the intracellular signaling. Lastly, knockout (KO) mouse for either of the isotypes showed relatively weak effect on B cell development indicating that IgM and IgD could compensate for each other's function (34–36). Thus, IgD was thought to be a reserve receptor to ensure functional immune responses as IgD-BCR may activate B cell signaling in case IgM-BCR fails. However, recent studies provide compelling evidences indicating functional segregation of IgM and IgD on the B cell surface. As discussed below, several lines of evidence support this view.

### Altered B Cell Development

**Figure 1:**
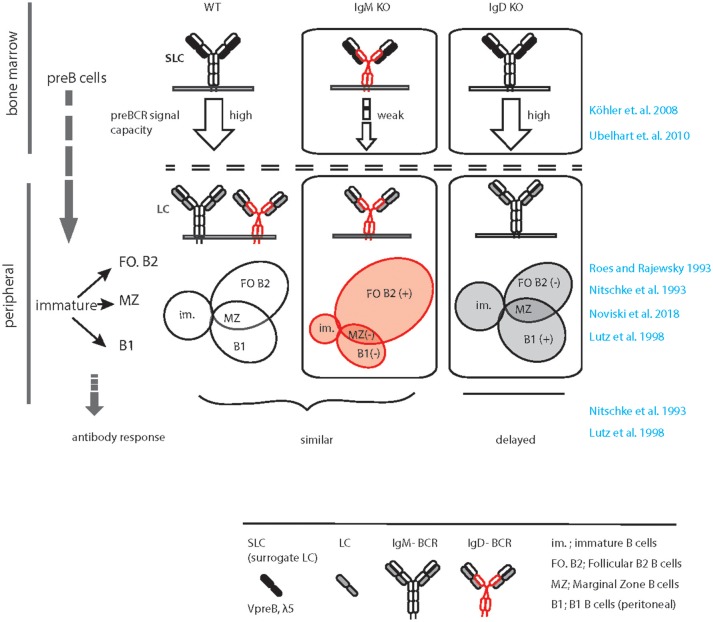
Graphical summary of altered peripheral B cell subsets and immune response in IgM KO and IgD KO animals. Schematic representation of B cell development in WT (left), IgM KO (middle), and IgD KO (right) animals highlighting the defect in preBCR signaling, increased follicular (FO) B cell compartment and decreased B1/ MZ B cell compartment in IgM KO animals. In contrast, follicular (FO) B cell compartment is reduced and B1/ MZ B cell compartment is increased in IgD KO animals. In response to antigen, IgD KO animals show a time delay and decreased serum antibody titer compared to WT animals.

In the wild type situation, IgM, but not IgD, efficiently associates with the germline encoded surrogate LC composed of VpreB and λ5 to form the pre-BCR, which is expressed at the pre-B cell stage of development (37, 38) (Figure 1). Expression of the pre-BCR triggers LC gene recombination driving B cell development further to the immature stage. Notably, the signaling capacity of the pre-BCR largely relies on μHC rather than on δHC that are usually part of IgM or IgD, respectively (Figure 1). At immature stage, differential poly-adenylation and alternative splicing of the Ig HC pre-mRNA encompassing the recombined V_H_DJ_H_ exon and the downstream *IGHM* (Cμ) and *IGHD* (C∂) exons lead to co-expression of IgM and IgD (6, 39). Such developmental regulation of IgM and IgD expression points to a specific non-redundant role of IgD in B cell development or function. In line with this, IgM KO mice (expressing only IgD) show a decrease in the proportion of innate-like B1 B cells that are known to require stronger signaling for their development and to express higher IgM and less IgD than follicular (FO) B cells (40). The marginal zone (MZ) and FO B cells seem to develop normally in the IgM-deficient mice although the number of FO B cells is slightly increased in these mice (33, 35) (Figure 1). In contrast, IgD deficiency results in mild effects in B cell development such as a decrease in the number of FO B cells to variable extent in different animals (34, 36) (Figure 1). Strikingly, IgD deficiency delays affinity maturation of B cells in primary antibody response against protein antigens (36) (discussed in section GC response and affinity maturation).

Usually, IgD expression begins at the transitional stage between immature and mature B cell compartments (6). As discussed before, alternative splicing of the HC pre-mRNA containing the Cμ and C∂ exons result in co-expression of IgM and IgD-BCRs. Such co-expression is associated with gradual decrease in IgM expression in mature naïve B cells as compared to immature B cells. Generally, immature B cells express increased amount of IgM while mature naïve B cells express increased amount of IgD together with slightly reduced level of IgM. However, B cells from IgM KO mice express almost 1.5–2.0-fold excess of IgD-BCR on the PM (33). On the contrary, IgD deficiency does not greatly increase IgM expression, although it limits the down-modulation of IgM expression within the mature B cell repertoire. Thus, in IgD KO mice all mature B cells tend to express increased amount of IgM similar to what is observed in wildtype immature B cells (Maity et al., unpublished data). Together, these alterations in IgM and IgD-BCR expression in the mature B cell compartments point to a potential difference in the strength of signals generated through these two BCR isotypes. It is conceivable that the threshold for survival signal is achieved by relatively low amount of IgM-BCR, but high amount of IgD-BCR on the surface is required. Thus, considering the role of BCR-induced signals in selection and survival of B cells, it is tempting to speculate that the signaling through IgM-BCR is stronger than that of the IgD-BCR.

In line with this hypothesis, it has been suggested that increased IgD expression in mature B cell compartment is necessary to down-modulate the increased amount of IgM on immature B cells, thereby setting a variable range of IgM expression across the B cell repertoire (33). Moreover, IgD-BCR induces mild signaling *in vivo* (33) despite being strongly signaling *in vitro* (41) and functionally equivalent to IgM *ex vivo* (unpublished result Maity et al.). Taken together, the elevated amount of IgD-BCR is necessary to enable the survival of IgM-deficient mature B cells, while IgD-deficient mature B cells achieve the signaling threshold required for survival by evading the down-modulation of IgM-BCR. Therefore, IgM expression on mature B cells from IgD-deficient mice is similar to that of the wildtype immature B cells. This suggests that the amount of IgM expressed on immature B cells is sufficient for selection and survival. Furthermore, the expression of IgD-BCR is not necessary for these processes and is most likely required for efficient mature B cell function.

### Selective Antigen Responsiveness

Another line of evidence for diverse functional specificity of IgM and IgD comes from their structural differences (32, 36, 42). The N-terminal V_H_ and V_L_ of HC and LC together form the antigen-binding site. The V_H_/V_L_ and the first constant domain of both HC and LC (C_H_1, C_L_) constitute the Fab (fraction antigen binding) fragment, which is joined with the next C_H_ domain through a hinge region. The structure of the hinge region is strikingly different for IgM and IgD (32, 36, 42). While IgM has a smaller hinge region, IgD is characterized by a long hinge region with charged residues and O-linked glycosylation (24, 42). The long hinge region gives IgD more flexibility to orient its antigen binding Fab fragment toward potential antigens. Thus, despite equal binding of monovalent and multivalent antigens, IgD is optimized for responding to multivalent antigen in immune-complexes (36, 42). Using an *in vitro* reconstitution system in SLP65, Rag2, and λ5 triple knockout (TKO) pro B cells, it was demonstrated that IgD-BCRs, specific for hen egg lysozyme (HEL), or 4-hydroxy 5-Iodo 3-nitrophenylacetyl (NIP), bind and initiate calcium response only to multimeric antigen complexes but not to monovalent antigens (32). In contrast, when expressed as IgM, the same BCRs were found to be responsive to monovalent antigens as well as to multivalent complex antigens. Swapping the hinge region between IgD and IgM also interchanged their specificity toward antigen valency. Remarkably, in the same study it was also shown that anergic B cells characterized by elevated surface IgD:IgM ratio failed to respond to monovalent antigens but remained fully responsive to multivalent complex antigens. Thus, higher IgD expression by anergic B cells is a mechanism to keep them quiescent toward monovalent autoantigens thereby preventing autoreactive responses, while they remain fully active against multivalent foreign immune-complexes thereby mounting proper immune responses. Conversely, higher surface IgM and low surface IgD expression in B1 B cells may allow activation by self-structures, which might be necessary for the house keeping functions of B1 B cells such as removal of cell debris. Simultaneously, B1 B cells retain the capacity to promptly mount innate immune response against common microbial antigens.

Similar evidence is also obtained from a recent study employing a transgenic reporter mouse in which B cell activation is monitored by green fluorescent protein (GFP) expression under the control of *Nr4a1* (Nur77), an immediate early response gene of antigen receptor signaling (33, 43). Using this model, the authors showed that IgD is less efficient than IgM in sensing endogenous antigens. While both isotypes can efficiently mediate GC entry, B cells lacking IgM are defective in differentiating into short-lived plasma cells (SLPC) (33). It is therefore conceivable that lowering surface IgM expression on mature FO B cells provides an important mechanism to limit their differentiation to antibody secreting SLPC thereby preventing uncontrolled immune responses to cross-reactive autoantigens that bind at low specificity.

### GC Response and Affinity Maturation

The germinal centers (GCs) are densely packed cellular domains within the lymphoid organs that are formed during the immune response (44). Within GC, antigen-specific B cells are selected, enriched and their antigen-binding specificities are improved in a process known as affinity maturation by SHM (13, 45). Since CSR also takes place in GC, the GC reaction is important for the generation of high-affinity antibodies with different effector functions including memory responses (46, 47). During T cell-dependent (TD) immune response, it is believed that a considerable number of FO B cells participates in the GC reaction (44, 48). As such, IgM deficiency neither significantly impacts the development of FO B cells nor it impairs TD-immune responses or affinity maturation through the GC reaction (35). In IgM KO mice, the TD immune responses toward carrier-conjugated monovalent antigens such as 2,4-Dinitrophenol-ovalbumin (DNP-ova) and complex antigen like sheep red blood cell (SRBC) remain unaltered as compared to wildtype counterpart (33, 35). However, a recent study (33) reported that the early class-switch response is defective in IgM-deficient B cells resulting in impaired generation of short-lived IgG1^+^ plasma cells (SLPC), although the unswitched PCs remained unaltered. Interestingly, this impaired IgG1^+^ SLPC is intrinsic to IgM-deficient B cells and independent of monoclonal or polyclonal antigens used for immunizing the animals (33).

Unlike IgM deficiency, studies using IgD-deficient mice revealed that absence of IgD leads to the retardation of TD-immune response, antibody production, and affinity maturation as compared to wildtype counterpart (34, 36). In particular, immunization with small-molecule antigens such as DNP-ova or NIP-chicken gammaglobulin (NIP-CG) showed a delayed antibody production and defective affinity maturation in IgD-deficient mice. Although the amounts of different serum Igs, except IgE, were normal in non-immunized IgD-deficient mice, antigen-specific IgG1, and IgG2 serum titers were largely reduced upon immunization (34, 36). As already mentioned, IgD-deficient mice also showed a delay in affinity maturation, i.e., production of high affinity antibodies, against NIP-CG by 3–4 days. On the contrary, using SRBCs as antigen, it was shown that IgD is redundant for GC reaction and immune response (33). Of note, unlike carrier conjugated small-molecules like DNP-ova and NIP-CG, SRBC is a robust polyclonal antigen, which can mount immune response independent of adjuvant. Together, these results suggest that the IgD-BCRs may be required for recruitment of B cells into GC reaction and subsequent affinity maturation during primary immune responses (34, 36).

Notably, studies employing mouse models of autoimmunity revealed that IgM-BCR exaggerates autoantibody production specifically in the absence of IgD (33, 49). For instance, the IgD-deficient lpr mice, a mouse model of systemic autoimmunity, showed elevated production of all different subtypes of IgG (IgG2a, IgG2b, and IgG3) autoantibodies, increased deposition of immune complexes in the kidney and more severe phenotype compared to IgD-sufficient lpr mice (49). Although these mice have elevated abnormal CD4^−^CD8^−^–double negative T cells in the spleen and lymph nodes, the severe autoimmunity in IgD-deficient lpr mice suggests a protective role for IgD-BCR in preventing deregulated autoimmune responses induced by IgM-BCR. In line with this, deficiency of IgD in Lyn^−/−^ mice, a commonly used model of systemic Lupus Erythematosus (SLE) also shows enhanced autoantibody production (33). On the contrary, IgM deficiency in Lyn^−/−^ background abrogates this autoantibody generation.

Taken together, the positive effect of IgM on early class-switch and on the generation of SLPC suggests that IgM-BCR may readily induce immune responses to autoantigen and that the presence of IgD-BCR negatively regulates this by attenuating the differentiation of autoreactive B cells into antibody secreting plasma cells. This view is in agreement with the lower threshold for activation of IgM as compared to IgD and with the fact that IgD binds to, but is not activated by, soluble monovalent antigens. Notably, an increased amount of monovalent antigen prevents the activation of IgD-BCR by immune complexes of the same antigen suggesting that IgD-BCR is regulated by the ratio of monovalent to complex antigen (32). Thus, all forms of antigens including autoantigens readily activate the IgD-deficient B cells that express only IgM. Most likely, the selectivity of IgD-BCRs toward antigen complexes and its regulation by soluble monovalent antigens controls the threshold for activation of wildtype B cells (42). Thus, similar to malignant transformation, the manifestation of autoimmunity may be a multistep process, in which the loss of IgD-mediated control together with the loss of a negative regulator, such as Lyn, result in rapid development of autoreactive immune responses. Thus, maintaining IgD at a higher proportion as compared to IgM may well be an important step in the prevention of aberrant outbreak of autoreactivity in wild type animals.

## Characterization of BCR Nanoclusters

The above discussion underlines the notion that characterizing the molecular mechanisms of BCR activation is critical for understanding B cell selection, survival as well as abnormal B cell responses toward autoantigens. While it is well-known that, upon binding the cognate antigen, the BCR activates B cell signaling and mediates antigen internalization (50, 51), the mechanism of signal initiation upon antigen binding remained a long-standing debate. Alternate models proposed an antigen-mediated cross-linking of adjacent BCRs or antigen-induced conformational change and rearrangement of BCR clusters (31, 52–54). There are experimental evidences both favoring and opposing these models, which have been reviewed elsewhere (54–56). Intriguingly, for all these models it is necessary to consider the initial state of the BCR prior to antigen binding, which remained a challenge for some time. The ordered assembly of the BCR on the PM in the native state was far below the resolution of confocal microscopes and therefore remained elusive (55). Recent advancement of microscopic techniques, especially the super-resolution techniques enabled the visualization of the nanometer scale organization of receptors on the PM (31, 57–59).

### Identifying BCR Nanoclusters by dSTORM

The most commonly used method for visualizing the nanoscale organization is the direct stochastic optical reconstitution microscopy (dSTORM). This method exploits the sparse blinking property of the fluorophores under reducing chemical environment combined with high energy excitation leading to dark state of the fluorophores (60). This induced stochastic optical blinking is recorded with high-speed acquisition system, usually an EM-CCD camera, which ensures splitting, and registering of fluorophore peaks or optical point spread functions (PSFs) into different frames. Finally, the individual frames are computed to obtain the ensemble high-resolution images. Resolutions of a dSTORM image are combined with the efficiency of correctly identifying the PSFs and localizing them with an empirically determined uncertainty (61, 62). Several factors including the samples, their preparation, types of fluorophores, performances of acquisition devices and relative drifts associated to microscope platforms during imaging influence the uncertainty of localizing the PSFs, which in turn determines the resolution. In practice, one could expect a 10-fold improvement of image resolution compared to standard fluorescence microscopy (31, 59, 63).

In recent years, dSTORM was employed by different laboratories to investigate the organization of BCRs on the PM in resting and activated B cells (31, 58, 59). Despite their differences in methods of sample preparation and sample source, the data obtained from dSTORM revealed that the native BCR resides as nanoclusters or protein-islands, and not as individual freely moving entities on the PM.

However, the mechanism of BCR activation by antigen-mimicking anti-BCR antibodies or antigen-independent cytoskeleton remodeling induced by Latrunculin A (LatA) remained controversial in these studies (31, 58, 59). The reasons for these variations are not completely understood and it is conceivable that they are linked to differences in methods and reagents as discussed below.

### dSTORM Imaging of Resting and Activated BCRs

**Figure 2:**
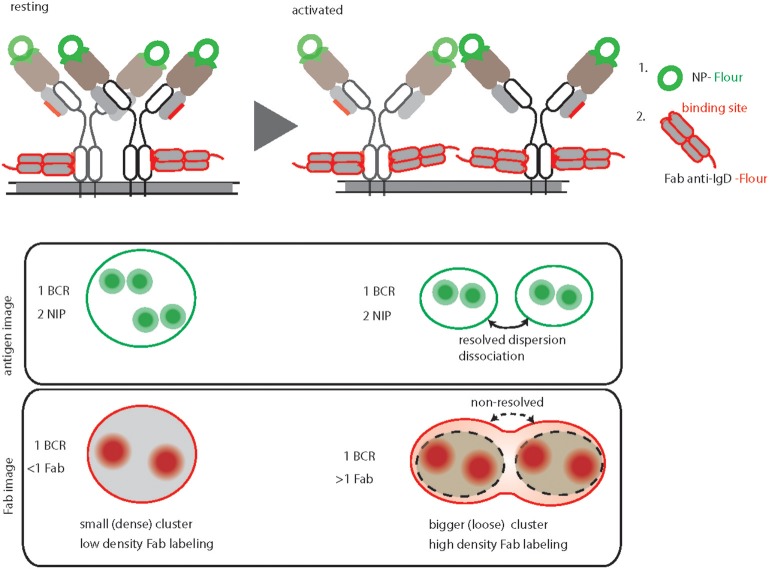
Graphical summary and comparison of antigen-based and anti-BCR Fab fragment-based BCR labeling for dSTORM imaging. Top, schematic representation of IgD-BCR nanoscale organization on resting **(left)** and activated **(right)** B cells, demonstrating their equally efficient fluorescent labeled NP-antigen (green) binding in contrast to differential fluorescently labeled anti-IgD Fab fragment (red) binding. The accessible sites for antigen and anti-BCR antibody (anti-IgD Fab fragment) of a NP specific IgD- BCR are highlighted by green and red color, respectively. Bottom, schematic of antigen-based dSTORM imaging compared to Fab fragment-based dSTORM imaging of resting and activated IgD-BCR nanoscale organization, allowing quantification of dissociated BCR units upon activation and non-resolving large clusters of activated BCRs due to increase labeling density, respectively.

In order to image the native organization of BCRs on the PM, every protocol must ensure non-stimulatory conditions and avoid induced clustering or crosslinking. To accomplish the non-stimulatory conditions, the labeling reaction should be performed on ice by using fluorescently labeled probes against the BCRs (31). Additionally, the probes must have equal labeling efficiency and accessibility to potential binding sites on different nanoscale structures starting from monomers to large oligomers (Figure 2). This is strikingly different for antigen based labeling as compared to anti-BCR antibody based labeling. Since antigen-binding is the main role of a BCR, the antigen binding sites are protruded on the top of the BCR molecule. Therefore, fluorescently labeled antigens would equally access and bind to the BCRs regardless of their dense or loose clusters (Figure 2, resting and activated). Indeed, the overall BCR density obtained by dSTORM for both resting and activated B cells remained consistent upon labeling with antigen (31). In contrast, the epitopes for the anti-BCR Fab fragments might be partially buried in BCR oligomers and more accessible for labeling only in dispersed BCR clusters or monomers (Figure 2). Therefore, labeling with Fab fragments might not identify the dissociation of BCR molecules from tight oligomers to dispersed and dissociated smaller units. Instead it detects dispersed smaller units with high density labeling making them indistinguishable from tight oligomers (Figure 2).

The number of fluorophores per cluster is used to deduce the number of BCR molecules within a particular nanoscale domain. This requires a defined staining and fluorophore labeling protocol, which can be linearly correlated to the number of BCR molecules. In the case of antigen staining, the BCR to antigen ratio always remains close to a 1:2 ratio (Figure 2) (31). Unlike small molecules (e.g., NP) or small proteins (e.g., HEL, MW 14.2 kDa), estimating the number of fluorophores attached to a bigger protein molecule such as Fab fragment antibody (MW 50kDa) is somewhat challenging. In addition, staining with Fab fragments generated from a polyclonal antibody is incapable of reporting a linear BCR to fluorophore ratio due to multiple binding sites of the antibody (58, 59).

The protocol of adhering B cells must guarantee the non-stimulatory conditions including native PM organization and preferably untouched receptors. In this regard, settling cells at low temperature (on ice) extensively prevents activation and reorganizations (31). In contrast, adhering cells at 37°C or room temperature (RT) might induce altered membrane organization or BCR internalization resulting in acquisition of intracellular fluorescence during imaging, which may not be excluded by total internal reflection fluorescence (TIRF) mode microscopy (58, 59). Furthermore, activation of surface-adhered B cells by treatment with either anti-BCR antibody or LatA might influence the PM for induced changes and constrain the BCR dynamics (64, 65). Particularly, an induced crowding of the BCR toward the attachment surface seems to be unavoidable when using specific tethering agents to adhere B cells and to simultaneously activate B cells. In contrast, a simplified protocol to stimulate B cells followed by staining, attachment and fixation avoids any further distortions as well as provides opportunities to compare with other microscopy methods (31, 66).

In summary, the dSTORM technology provided evidence for nanoscale protein-islands organization of the BCR in the resting state. However, characterization of the active state of BCRs by dSTORM method still remains challenging. Due to lack of a consensus protocol, it is difficult to compare among different studies. Therefore, the application of super resolution imaging for BCRs or any other immunoreceptors must be updated and rationalized to visualize the native membrane organization.

### Isolated Nanoclusters of IgM and IgD-BCRs

Intriguingly, the evidences that BCR molecules are organized in nanoclusters inspired new model of isotype-specific segregation of IgM and IgD on the PM of resting B cells. In turn, two-color dSTORM experiments facilitated the visualization of IgM and IgD-BCRs simultaneously and revealed their independent nanocluster organizations in separate membrane domains (31). Moreover, the size and number of receptors per nanoclusters of IgM and IgD are strikingly different from each other (31, 59). While IgD nanoclusters contain approximately 48 BCRs within a radius of about 240 nm, IgM nanoclusters contain 30 BCRs within a radius of about 150 nm (31, 42). This difference in size is proportional to the relative expression of BCR isotypes on the cell surface, which is usually in the ratio of 65 to 35 for IgD and IgM-BCRs, respectively. In addition, two-color dSTORM also reported an average distance of 300–350 nm that separates individual nanoclusters of IgM and IgD-BCRs (31, 42). This was further supported by two-marker electron microscopy imaging of the B cell PM (31, 42). Notably, the differences in the number of receptors per nanoclusters were also reported in single-color dSTORM using anti-BCR Fab labeling protocol, but the separation between IgM and IgD-BCRs could not be measured by this method (59).

The fact that IgM and IgD BCRs reside in separate membrane domains supports earlier biochemical studies revealing that they can be stimulated independently from each other (67). Indeed, using two-color dSTORM it was shown that the stimulation of one isotype of BCR did not impact the organization of the other isotype, thereby facilitating their independent signal initiation (31, 55). Furthermore, IgD-BCRs reside in lipid raft-like membrane domains and co-localize with glycosyl-phosphatidyl-inositol linked (GPI) protein CD52, while IgM-BCRs reside in non-raft domains prior to activation (68). The raft-like membrane domains are rich in GPI protein, GM1 gangliosides, and other important co-receptors such as CD19 and CXCR4 (42, 69, 70). Indeed, IgD-BCRs co-localize with CD19 and CXCR4 in resting B cells, whereas IgM-BCRs gain proximity with CD19 only upon activation. Interestingly, both CD19 and CXCR4 are considered to be co-activators to BCR signaling, while the receptor phosphatase CD22 acts as an inhibitor to BCR signaling. Notably, CD22 also exists as preformed nanoclusters and its proximity increases with IgM-BCR upon activation (71, 72). These observations are in full agreement with the fact that the translocation of BCRs into lipid rafts is necessary for stronger signaling (73) and that the signaling through IgM-BCR differs from IgD-BCR. In the next section we discuss how the interplay between CXCR4 and BCR isotypes modulates B cell function in healthy and neoplastic condition.

## Synchronization Effect of Chemokine Receptor CXCR4

### CXCR4 Signaling in Healthy and Malignant B Cells

CXCR4 belongs to the G protein coupled receptor (GPCR) family that selectively binds the CXC family chemokine Stromal Cell-Derived Factor 1 (SDF-1) also known as CXCL12 (74, 75). In response to CXCL12, CXCR4 signaling activates diverse GPCR pathways, resulting in migration, adhesion, and transcriptional activation of downstream target genes. Interestingly, recent findings uncovered functional differences between IgM and IgD BCR isotypes in the context of CXCR4 chemokine receptor signaling (76). IgD-deficient B cells were found to be defective in CXCR4 signaling with no calcium mobilization upon CXCL12-mediated stimulation of CXCR4 and impaired chemotactic migration toward CXCL12 gradient. In contrast, IgM-deficient B cells, expressing only IgD B cells showed no impairments in CXCR4 signaling. Interestingly, stimulation of the co-receptor CD19 with anti-CD19 antibody restores the above-mentioned defects in IgD-deficient B cells. Deeper inspection led to the observation that CXCR4 and CD19 co-localize in the same nanocluster as IgD on the cell membrane but not within IgM nanoclusters (55, 76). Thus, physical separation of these two isotypes on the B cell membrane also implies their functional specificity (42).

In addition to the prominent role of CXCR4 signaling in pro and pre–B cells in the bone marrow, this chemokine receptor also controls the migration of mature B cells into secondary lymphoid tissues (77). Therefore, CXCR4 signaling is of particular importance for lymphomagenesis, infiltration, migration, and retention of leukemic B cells in particular lymphoid tissues (78, 79). For instance, a number of studies pointed out an important role of CXCR4 signaling in Chronic Lymphocytic Leukemia (CLL) (77, 79). CLL is a mature B cell malignancy characterized by clonal accumulation of CD5^+^ B cells in peripheral blood, bone marrow and secondary lymphoid organs (80, 81). Continuous BCR signaling is considered to be the central pathway mediating the pathogenesis (82–84). It has been shown that ligation of surface IgM-BCR by anti-IgM antibody leads to B cell signaling in CLL, while IgD ligation by similar antibody treatment is unable to activate these cells (85, 86). Interestingly, stimulation using anti-IgM antibodies reduced the chemotaxis of CLL B cells toward CXCL12, while IgD stimulation led to opposite result suggesting IgD dependence of CXCR4 signaling (77, 87).

Often, expression of CXCR4 in neoplastic B cells in CLL is enhanced compared to normal B cells thereby conferring increased functional response to CXCL12. Indeed, CXCR4 overexpression in these neoplastic B cells is regarded as one of the factors responsible for their enhanced migration toward bone marrow niche, enriched with stromal cell derived CXCL12 (77, 83, 88). Furthermore, increased CXCR4 expression on CLL cells also accounts for their resistance to spontaneous or drug-induced apoptosis, providing a protective niche for tumor cells, and making them unresponsive to conventional chemotherapy (79, 88–91).

Apart from deregulated expression, several CXCR4 mutations are common to leukemic B cell and related disorders including CLL and Waldenström macroglobulinemia (WM) (92–94). The most interesting CXCR4 somatic mutations are truncations of the C-terminal tail by 9–12 amino acids. This is also the commonly found germline variation in warts, hypogammaglobulinemia, infections, and myelokathexis (WHIM) syndrome. WHIM-like CXCR4 mutation results in CXCL12 desensitization and sustained CXCR4 signaling in leukemic cells (95), which manifests in the clinical warts-like symptoms in WHIM patients. Furthermore, the WHIM-like CXCR4 mutation accounts for sustained survival signal in leukemic cells and renders them resistant to inhibitors of the Bruton's tyrosine kinase (BTK) (96). Interestingly, BTK is a downstream kinase of BCR, Toll-like receptor (TLR), and CXCR4 (97). BTK inhibition causes impaired CXCR4 signaling and reduces the PM pool of CXCR4, resulting in rapid egress of CLL cells from CXCL12-rich niches and consequently prevents re-entry of CLL cells (78, 98, 99). Thus, simultaneous deactivation of both BCR and CXCR4 signaling reveals the clinical efficacy of BTK inhibitor, demonstrating the complex interplay between BCR and CXCR4 (100, 101).

### CXCR4 and Cytoskeleton Remodeling

**Figure 3:**
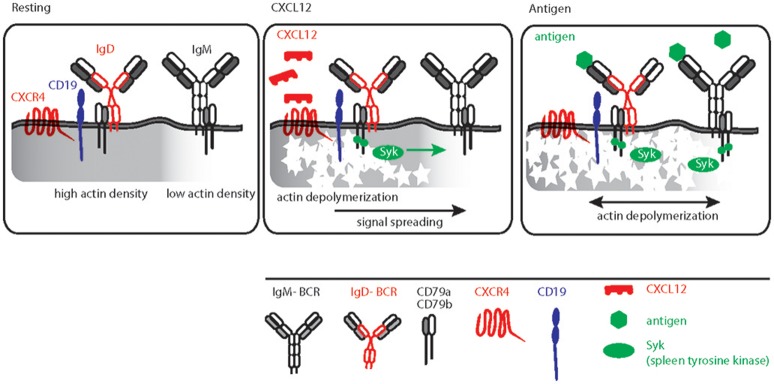
Schematic representation of CXCL12 and antigen triggered B cell activation. Left, resting B cell membrane showing IgD and IgM BCR nanoclusters differentially localized in actin dense (gray shaded) and actin poor regions, respectively. IgD BCRs are residing in close proximity to CD19 and CXCR4. Middle, CXCL12 induced signaling in B cells depicting the sequential triggering of local actin remodeling, recruitment of Syk and IgD BCR activation followed by signal spreading toward actin poor domains. Right, antigen mediated BCR signaling, Syk recruitment and actin remodeling leading to massive actin remodeling and B cell activation.

Similar to other GPCRs, CXCR4 signaling also induces actin cytoskeleton remodeling (76, 102). This links CXCR4 signaling directly to BCR signaling as actin depolymerization by either LatA or CytoD is sufficient to induce robust BCR signaling (31, 65, 103). Furthermore, B cell-specific loss of function mutations in actin binding protein and related regulatory molecules (ABP-1 and WIP) modulates BCR signaling confirming pivotal role of the actin cytoskeleton for signal initiation and processing (50, 104–106). Although the molecular basis for actin cytoskeleton and BCR signaling cross-talk was mechanistically interpreted by picket-fence model, the *in vivo* trigger of this axis remained elusive. Increasing evidence suggests that the dominant IgD isotype on mature B cells is proximal to CXCR4 and therefore, IgD is required for CXCR4 signaling in mature B cells (Figure 3). This is in line with the observation that, in comparison to the IgM-BCR, the IgD-BCR resides in more actin-dense regions of the PM (Figure 3) (65). Thus, the effect of releasing the BCR from the constraints posed by the actin picket-fence might be greater for IgD than for IgM, although the exposure of B cells to LatA results in the dissociation of BCR oligomers of both classes, IgD and IgM (Figure 3) (31, 68). Together, the differential association of BCR isotypes with chemokine receptors confirms the functional specificity of IgD-type BCR and its role in efficient integration of the migratory cues from lymphoid tissue environment and antigen recognition during an immune response. In parallel, cooperating with CXCL12 induced CXCR4 signaling, IgD ensures low-grade activation of mature B cells in absence of antigen (Figure 3).

## Conclusion

The existence of different classes of antibodies and their BCR counterparts in mammals is certainly related to their evolutionary conservation and necessity to diversify the repertoire. Nevertheless, the prominent usage of IgM and IgD during B cell development mark them as specialized antigen receptors compared to other isotypes. Overcoming the previous redundancy postulate, we begin to understand functional difference between IgM and IgD. With its structural specificity for multivalent antigens, its isolated nanocluster membrane organization and its coordination with particular co-receptors, the IgD BCR regulates and diversifies B cell responses. However, much more remains to be explored, specifically regarding the role of IgM and IgD in neoplastic B cells and autoimmune diseases. The interplay between CXCR4 and BCR isotypes in leukemic cells and their impact on pathogenesis also remains of particular interest.

## Author Contributions

PM, MD, and HJ reviewed the literature and wrote the manuscript. PM and MD prepared the figures. AN contributed to the writing of the CXCR4 segment. All authors read and approved the review.

### Conflict of Interest Statement

The authors declare that the research was conducted in the absence of any commercial or financial relationships that could be construed as a potential conflict of interest.
